# Land use classification using multi-year Sentinel-2 images with deep learning ensemble network

**DOI:** 10.1038/s41598-025-12512-7

**Published:** 2025-08-08

**Authors:** J. Jagannathan, M. Thanjai Vadivel, C. Divya

**Affiliations:** 1 School of Computer Science Engineering and Information Systems, Vellore Institute of Technology, Vellore, Tamil Nadu India; 2 Department of Computer Science Engineering, Nandha Engineering College, Erode, Tamil Nadu India; 3 Centre for Information Technology and Engineering, Manonmaniam Sundaranar University, Abishekapatti, Tirunelveli, Tamil Nadu India

**Keywords:** Artificial Intelligence, Deep learning, InceptionResNetV2, IRUNet, Land use classification, Remote sensing, Satellite imagery, Sentinel-2, Test-time augmentation, UNet, Climate sciences, Environmental sciences, Natural hazards, Engineering

## Abstract

Accurate land use classification is essential for urban planning, environmental monitoring, and agricultural management. Sentinel-2 satellite imagery provides rich spatial and spectral information suitable for this purpose. This study proposes a deep learning ensemble network named IRUNet, which integrates InceptionResNetV2 with a UNet framework for multi-year Sentinel-2 imagery classification over the Katpadi region (2017–2024). Unlike prior works, IRUNet utilizes multi-scale feature fusion and incorporates Test-Time Augmentation (TTA) to enhance prediction robustness. While the data spans multiple years, each year is treated as an independent input without modeling temporal sequences. The proposed method demonstrates superior performance over UNet, ResUNet, and Attention-UNet models, achieving an accuracy of 98.21% and Dice similarity coefficient (DSC) of 88.96%. Additional metrics including precision (94.71%), recall (89.19%), F1-score, and Kappa coefficient have been reported. This research contributes a high-performance, generalizable framework for multi-year land use classification.

## Introduction

In today’s rapidly changing world, it is crucial to prioritize detailed land use classification^1^. Monitoring land use changes is critical in the context of rapid urbanization, environmental degradation, and sustainable development. Remote sensing with Sentinel-2 imagery offers high-resolution multi-spectral data ideal for land use and land cover (LULC) classification.

However, several technical challenges persist: Spectral similarity between classes (e.g., bare land and urban areas). Spatial complexity and scale variability. Inconsistencies across time (seasonal/temporal changes). Traditional machine learning approaches often struggle to handle these challenges, necessitating more robust, deep learning-based methods^2^. The capability of a deep learning-based ensemble network is a very powerful tool for land use classification. Especially for a country like India that is experiencing migration to its towns and cities as well as economic development, the understanding of these structures becomes important from the perspective of societal sustainability.

The research was conducted in Katpadi region, a fast-growing area within the Vellore District of Tamil Nadu, with a particular focus on the local vicinity surrounding VIT (Vellore Institute of Technology). Despite the region being a hotspot for urban expansion, agricultural practices and environmental management are all major drivers of land use change globally. Two states, Andhra Pradesh and Karnataka, surround this district. Katpadi, a city, is experiencing urbanization as a result of the increasing population.

Over time, satellite remote sensing has proven to be a very useful tool in monitoring and analyzing land use changes. New machine learning techniques mean that we are now able to process large amounts of satellite imagery and categorize it effectively. Among these techniques, CNNs and their family, such as DenseNet, have proven to be powerful methods for image classification. DenseNet, also known as Dense Convolutional Network, excels in deep learning with minimal parameters and effectively addresses the vanishing gradient problem, rendering it a superior option for complex satellite images. The ensemble network encompasses cutting-edge, large-scale architectures like InceptionV3 and VGG16, honed to identify pests and diseases affecting rice^1^. The impact of deep learning, in conjunction with other machine learning techniques, has been revolutionary for the field of Earth observation and remote sensing. The sophistication of its use cases has transformed the study into machine vision research, data fusion, object detection, and enabling semantic segmentation. Meanwhile, it can also aid in the advancement of other engineering fields, such as 3D reconstruction and large-scale monitoring^3^.

In addition, deep learning is responsible for handling big data and identifying complex patterns in the datasets^1^. Researchers have utilized LiDAR networks for multiscale semantic feature optimization and fusion in high-resolution networks. Yuan et al.^2^. Furthermore, the advancements in deep learning and the abundance of geological data for exploration have significantly enhanced our capacity in 3D conceptual construction, as noted by Deng et al.^3^. Shah et al. They conducted a comparison between CNN, VGG 16, VGG 19, Inception V3, and ResNet 50, utilizing rice data^4^. Stoian et al.^5^ suggested another important factor: the ability of our deep learning-based ensemble network to grow. This is particularly crucial when dealing with extensive time-series Sentinel-2 data, as it enables continuous monitoring and analysis of changes in land use. By building the ensemble network, our method can take full advantage of convolutional neural networks (CNNs) to extract visually spatial and temporal information from the Sentinel-2 image time series proposed by Teyou et al.^6^. This allows us to create precise, up-to-date maps of what kind of thing covers most land in different places and over time. Cheng, Xinglu, et al. have proposed an approach that significantly benefits the research of remote sensing technologies and their application in land-use studies^7^. In summary, given the rapid changes in today’s world, it is crucial that we implement deep learning-based ensemble networks for time-series Sentinel-2 image land use classification. This will enable us to monitor and understand these phenomena more accurately, contribute to environmental conservation efforts, and support sustainable development. However, this study’s use of time-series Sentinel-2 represents a noteworthy innovation in the field of land use classification, employing deep learning-based ensemble networks. This innovation has implications for today’s fast-paced world, enabling accurate monitoring and understanding of LULC dynamics, their efficient absorption, utilization, or replacement, and the management of environmental restoration programs under sustainable development strategies using the provided information. Convolutional neural networks (CNNs) can generally learn features that take into account the context of pixels, which allows them to produce a better representation for such data^5^.

Recent advances demonstrate the efficacy of deep convolutional networks like UNet, DenseNet, and InceptionResNet for satellite image segmentation. Yet, they individually suffer from limitations such as insufficient multi-scale feature fusion or sensitivity to input perturbations.

### Motivation

To bridge these gaps, we develop an ensemble deep learning framework that integrates powerful feature extraction (InceptionResNetV2) with efficient pixel-wise segmentation (UNet), enhanced further by applying Test-Time Augmentation strategies.

### Major contributions

This study presents the following major contributions:

A novel ensemble architecture, IRUNet, combining InceptionResNetV2’s hierarchical feature extraction with UNet’s precise segmentation capability. Application of Test-Time Augmentation (TTA) to improve model generalization and robustness. Development of a comprehensive dataset covering eight land use categories across eight years (2017–2024) in the Katpadi region. Comparative evaluation against existing state-of-the-art models including UNet, ResUNet, and Attention-UNet. Analysis of multi-year land use dynamics in a fast-growing urban area.

## Literature review

Land use categorization to identify the type of land space in a region using multitemporal Sentinel-2 photos has been a popular study topic in recent years for environmental monitoring, urban planning, and agriculture management. To enhance the robustness and accuracy of classification, recent studies have included deep learning techniques such as ensemble networks. Only in recent years, thanks to substantial data processing, machine learning, and artificial intelligence developments, have the doors been opened. Convolutional neural networks have successfully introduced deep learning methods for HSI classification in recent years. Nevertheless, due to the high dimension of HSI data and few available training samples, overfitting is a common issue. Chhapariya et al.^6^ aim to solve this issue with DSSpRAN, which proposes a deep spectral-spatial residual attention network that takes advantage of one spatial and three spectrum scenes by utilizing the residual attention mechanism on spectral and spatial data. This method enhances the classification accuracy at both the spectral band and spatial relationship level of pixels by emphasizing features directly. Experimental results demonstrate that the DSSpRAN outperforms other state-of-the-art methods across various classifications of land use, with improvements generalized on a number of datasets. Wang et al.^7^, A new classification framework for bamboo mapping integrating in-situ, GEDI, and time-series Sentinel-2 images with a whale-optimized dual-channel Dense Convolutional Network (DenseNet). It has successfully dealt with the challenges of discriminating bamboo from other types of vegetation, providing top accuracy (90.81%), recall (91.86%), and F1-score (91.33%). The research highlights the value of integrating structural variables from GEDI, which improved mapping performance over conventional spectral information methods by > 5%. They were able to show that their revolutionary usage of dual-channel DenseNet (along with Drop Block regularization) can surpass traditional deep learning methods, even on complex terrains.

According to Peng et al.^8^, recorded applications of CNN-based pre-trained models for agricultural crop monitoring highlight the implication of climate change on ecosystems and the urgent need to address climate change and its effects. The studies used architectures such as VGG16, MobileNetV2, DenseNet121, and ResNet50 to improve classification from images that had been enhanced using the aforementioned enhancement profiles and fine-tuning methods. The findings highlight the potential of enhanced CNN models in remote sensing applications, particularly for accurate agricultural decision-making and resource management.

In line with precision agriculture, Albarakati et al.^9^, when it comes to analyzing remote sensing data for LULC classification, introduced a self-attention document CNN architecture. In particular, by combining self-attention mechanisms at the network level, their method greatly improved the accuracy of classification on the SIRI-WHU, EuroSAT, and NWPU datasets. Moreover, their quantum optimization method enhanced feature selection that resulted in a higher performance over a wide range of agricultural applications.

Zheng et al.^10^ proposed a multitemporal deep fusion network (MDFN) based on CNN for short-term and multi-temporal high-resolution (HR) image classification. Their method utilized a spectrum of CNN and long short-term memory (LSTM) networks to efficiently store spatiotemporal features. This network did better than other methods when tested on real-world multitemporal HR datasets. This shows that short-term satellite data could be used for accurate LULC mapping.

With combined data from multiple satellite sensors, Kumar and Gorai^11^ did a study to see how deep CNN and DNN compare for LULC classification. The results showed that CNNs outperformed DNNs in classifying mining zones, and the fused data approach further enhanced the classification accuracy. The results demonstrate the high effectiveness of CNN models when utilizing combined multi-source data to classify various land uses in challenging environments such as mining sites.

Rukhovich et al. have developed a novel method for SOM mapping (BRSD) that couples multitemporal soil line coefficients with neural network filtering of extensive remote sensing data^12^. Using data from 1984–2023, this approach auto-generates accurate SOM maps on arable land considering soil line spectral neighborhood (SNSL). We have deployed it for areas larger than 79,000 hectares in monthly cycles at a spatial resolution of 30 m on the territory of the Mtsensk district in Russia, employing neural networks to detect bare soil surfaces (BSS). Their analysis revealed a polynomial relationship between SOM content and soil line coefficient "C" with an R2 of 0.8. This method demonstrates the potential for use in SOM mapping across different soil types without time-consuming field surveys in transitional zones such as leached chernozems and sod-podzolic soils.

Xu et al.^13^ summarized the outcomes of the IEEE Geoscience and Remote Sensing Society Data Fusion Contest, 2018, with challenges on urban monitoring by multisensor optical remote sensing data. Given the amount of data, the competition explored state-of-the-art machine smart methods (such as CNNs) applied to integrate multispectral LiDAR knowledge with different knowledge sources (e.g., hyperspectral). These strategies, as the winners showed, were effective in demonstrating that CNNs can achieve greater than 80% accuracy for LULC classification, as well as highlighting the importance of multi-sensor data fusion and expert knowledge. The results encourage public availability of larger datasets for classification improvements and further investigation into data fusion, as well as sensor-dependent processing approaches.

Zheng et al.^14^ introduced a modified version of DeepLab V3 + with GauGAN data synthesis to address the issue of imbalanced land use datasets in remote sensing research. They focused on generating synthetic data to tackle the challenge of having few sample features, leading to higher classification accuracy. This allowed for stable training of the models through an attention optimization method, and spectrum normalization makes it easier to train a stable model, while improvements to the DeepLab V3 + architecture improved the use of high- and low-level semantic information merge. In terms of high-precision land use classification, this strategy did better than state-of-the-art baselines like U-Net and TransUNet. This shows that data augmentation and model adaptation are good ways to deal with problems caused by imbalanced datasets.

Huang et al.^15^ proposed daily urban land cover classification based on object-oriented deep learning through combinations of synthetically fused segment objects, deep features, and spatial association feature data. Using a synthetic semi variance function to optimize hyperparameters, the study showed that better image super pixel segmentation has a positive effect on classification results. This method combines a CNN for deep feature extraction with GCNs to capture spatial associations for classification using random forests. The results indicate that segment-based methods outperform traditional pixel-level approaches in achieving greater classification accuracy. The multidimensional feature fusion method can effectively fuse the advantages of single features to improve urban land cover classification efficiency.

Ismaeel and Kumar^16^ used multiple spectral indices and a DNN model to tackle the challenges for urbanization and built-up mapping. To define built-up areas, the study used indices such as the Normalized Difference Built-up Index (NDBI), Built-up Area Extraction Index (BAEI), and Normalized Built-up Area Index (NBAI). An innovative intersection strategy among these indexes enhances the mapping process, resulting in a final built-up map with 92.5% accuracy and a Kappa coefficient of only 0.848. Using Landsat 5 images, the trained DNN model predicted built-up regions with an accuracy of over 95%. The method proves to cope with misclassifications in dense regions, but performance for time-series analysis should be further evaluated, and so together with comprehensive training and validation protocols, it needs complex solutions for optimizing DNN.

Siqi et al.^17^ have estimated the urban land surface temperature (LST) using a hybrid model of Geographically Weighted Regression (GWR) and a deep neural network (DNN). This method advances thermal environment modelling to address the increase in urban heat problems. We then tested the hybrid model with other data-driven techniques on a sizable dataset containing 155,728 data points collected over four years in Hong Kong. The hybrid model yielded R2 values of 0.85 and 0.73, which were significantly higher than those of the OLS, GWR, and DNN models in both regions. The better performance of this model is because this model captured the geographical heterogeneity and considered the interdependencies of explanatory variables. It discusses the LST sensitivity to different factors and offers recommendations for controlling excessive urban heat, which can contribute to modelling the urban thermal environment and the components that determine changes in LST.

Li et al.^18^, Big Data and AI in Mapping Urban Landscapes: A Review of Deep Learning Algorithms for Urban Land Use Classification. Their tests in Shenzhen, China, investigated several deep learning settings and discovered that combining huge geospatial datasets with advanced algorithms can improve the accuracy, efficiency, and automation of land use mapping. This article first provides an overview of key challenges in urban land use classification research and then proposes avenues toward more widespread adoption of deep learning approaches in this field.

The design of Anitha et al.^19^ specifically targets the detection of long-range relationship features in remote sensing images. It adopts this bicolor architecture as RGB and HSV color spaces, which integrates a Residual Network-50 (ResNet-50) backbone with Downstream Lingering Feature Pyramid Network (DsLFPN) followed by Duo Transformer. Our tests with DsLATNet on different datasets showed that it did very well compared to the best systems currently available. It achieved higher overall accuracy for LULC classification by providing excellent classification accuracy metrics.

Ji et al.^20^ conducted a study on sea-land segmentation. This paper proposed E-Net, a lightweight convolutional neural network (CNN) with a novel E-shaped architecture that more effectively utilizes hierarchical features while remaining computationally efficient. To accurately segment complex sea-land boundaries, the proposed network introduces a new contextual aggregation attention mechanism (CA2M). The E-Net outperformed expectations, achieving mean Intersection over Union (mIoU) scores of 92.78% and 93.62% on two benchmark datasets. Its small processing cost also makes it a promising option for low-cost real-time applications. To address the inefficiencies that exist in previous EEG emotion identification models, Zhai and Guo^21^ proposed a novel lightweight adaptive dynamic focusing convolutional neural network (LAND-FCNN). LAND-FCNN effectively pulls out current features from partial data by using batch interactive attention and partial convolution (PConv). This helps avoid data shortages without the need for data enhancement. The suggested difference is in the use of Glance and Focus Dynamic Inference Networks (GFnet), which focus on important parts of tasks. This makes inference go faster and give better results across a range of datasets.

Zaabar et al.^22^ developed a hybrid convolutional neural network (CNN) and object-based image analysis (OBIA). We then developed a highly efficient LULC model using the aforementioned techniques and conducted a study in the Temouchent coastal region of Algeria. We employed high-resolution satellite images and CNN configurations to accurately predict segmented objects. With an overall accuracy of 93.5%, the results were promising, suggesting that we could implement the high spatial resolution genotype-trait inferences on a much wider scale to inform management and urban planning decisions.

El-Rawy^23^ applied the modified U-Net convolutional neural networks to evaluate and classify salt-affected soils around the Siwa Oasis, Egypt. They developed salinity maps and soil quality indicators using remote sensing data and deep learning algorithms. The new U-Net architecture, which added more salinity and vegetation variables, worked well for salinity detection, giving an accuracy rate of 91.27%. The burgeoning issue of soil salinization is reinforced here, emphasizing the need for technologically advanced monitoring methods.

Chowdhury^24^ looked at how well random forest, support vector machine, artificial neural network, and maximum likelihood methods worked for classifying LULC in Dhaka, Bangladesh. In analyses, the overall accuracy of 0.95 for ANN was consistently superior to that of other methods. It underlines the need for custom machine learning algorithms relevant at urban levels of complexity.

Alem and Kumar^25^ conducted an evaluation of deep learning models for remote sensing image classification. On their task of interest, they initially compared models trained from random initialization to pretrained (frozen) networks and found that fine-tuning provided a very large boost in classification performance. They find that deep learning systems are able to adjust for the unique characteristics of remote sensing images to enhance the speed and precision of LULC classification.

Acuna-Alonso et al.^26^ stressed the usefulness of CNNs in LULC identification and modeling, particularly in natural regions under special protection. Their findings demonstrated the CNN model’s ability to attain 91% accuracy on training datasets, proving the effectiveness of deep learning technologies in ecological evaluations and resource management.

Teyou et al.^27^ claimed that deep learning can automatically construct high-level spatial detectors from uninterpreted data, eliminating the requirement for artificial feature engineering. Large amounts of Sentinel-2 data could be processed thanks to the scalability of deep learning-based ensemble networks. This made it possible to monitor and evaluate changes in land use over a long period of time and on their own. Cheng et al.^28^ technique can overcome the disadvantages of existing categorization methods while also requiring many specific geographical time courses to fully comprehend land changes. Deep learning-based ensemble networks make monitoring land use changes easier and more efficient since they can learn from temporal variables in a wide range of time-series Sentinel-2 data. Furthermore, the combination of remote sensing technology and deep learning-based ensemble networks will enhance the capacity to support field observations and surveys for the widespread interpretation of land use and land cover change. This union enables the compilation of precise and current land cover maps, which can aid in the identification of alterations on Earth, such as sea-level rise, extreme weather occurrences, erosion, changes to ecosystems or resources, and so on. RNN and CNN are two prominent methods for extracting temporal and geographical information from multi-temporal data received through satellite observations. For example, Zhao et al.^29^ revealed how well the CNN-RNN hybrid model performed for land use classification while accounting for the temporal continuity of Sentinel-2 data.

Zhang et al. expanded on the strategy of Zhang et al.^30^, who applied a spatiotemporal attention module to improve the classification results of complicated land cover classes.

Furthermore, another strategy, including the use of self-supervised learning and transfer learning, has emerged as an effective tool for overcoming the challenges associated with a shortage of labelled data. In other words, we achieved amazing results by using pre-trained models on large data sets and fine-tuning them for a specific land use classification task. Yasir, Muhammad, et al. ^31^ suggested an ensemble strategy based on GNN that accurately modeled spatial relationships across Sentinel-2 time-series images while improving classification performance.

Zhang et al.^32^ provided a comprehensive evaluation of satellite remote sensing for LULC analysis, emphasizing the importance of multispectral and hyperspectral capacity in providing detailed land-cover information. The essay summarized the importance of high-resolution imaging in monitoring environmental changes and managing resources effectively. Mansour et al.^33^ used Sentinel-2 to examine urban sprawl in Ho Chi Minh City, highlighting the use of historical satellite data in urban research. The study emphasizes the importance of long-term data in understanding urban expansion trends and their implications for sustainable urban development. Dhanaraj et al.^34^, using Landsat and Sentinel, explore change in LULC over the Indian subcontinent. Image acquisition. They found that this dynamic nature of land-cover change in India warrants regional-scale studies for effective guidance of policies and land-use practices. Avcı and Cengiz^36^ implemented random forest classifiers for LULC and proved that random forest classifiers can properly work with big datasets and complex classification algorithms. The review is useful in terms of random forest applications and pointed out the necessity for classifiers. Li and Jiaxin^37^ consider the use of convolutional neural networks (CNNs) in deep learning for remote sensing applications. This research provides a context that explains the benefits of deep learning algorithms for image classification, a crucial step in LULC classification.

Huang et al.^38^ with this hypothesis: utilizing fewer parameters for composing deeper networks and enhancing the gradients flow. Hence, we chose this architecture as it has shown tremendous improvements in the challenging image classification tasks and is very well suited for satellite image validation. Zhao and Feng^39^ used DenseNet for high-resolution satellite image classification with significant accuracy improvements compared to conventional CNN architectures. Their research shows how DenseNet can get you one step closer to precise remote sensing applications.

Muthumperumal et al.^39^, using satellite photography, conducted an investigation on the hyper-urbanization of Chennai, Tamil Nadu. The research provides vital information on urbanization trends in the region and highlights the challenges of sustainable urban development against a backdrop of rapid growth. Arimjaya and Dimyati^40^ reviewed the impact of urbanization on agricultural land use changes in Tamil Nadu. Their findings highlight the need for development policies that balance urban expansion with similar attention to rural land use issues. In^41^, researchers implemented land use classification using VGG and compared it with historical data. In^42^, a hybrid model was proposed to predict climate changes, which can also be used with satellite data. Hamza et al. LULC recognition architecture with a bottleneck residual and self-attention fusion ^43^, which fuses features from neutrally synthesized satellite images. Using innovative techniques for feature extraction and optimization, impressive accuracy rates (99.0% and 99.4%) are reached on its system. In particular, it achieved the best performance compared to previous methods at the precision level and the redundancy data reduction level.

Based on the above survey Land use classification using remote sensing data has witnessed significant progress over the past decade, driven by deep learning innovations. Existing works can be broadly categorized into three groups:

1. CNN-based models for land use classification
  Convolutional Neural Networks (CNNs) such as UNet, FCN, and DeepLabV3 + have demonstrated powerful spatial feature extraction from high-resolution images. UNet’s encoder-decoder architecture with skip connections has become particularly popular for semantic segmentation tasks in Earth observation applications. However, standard CNNs are sometimes limited in capturing complex, multi-scale variations.
2. Time-series analysis in remote sensing
  Temporal modeling has been explored using architectures like ConvLSTM and Temporal Convolutional Networks (TCN). These methods capture seasonality and temporal dependencies across multi-date imagery. Nevertheless, most studies require dense, high-frequency temporal data, which may not always be available or consistent across years.
3. Ensemble methods
  Ensemble learning techniques that integrate multiple models (e.g., stacking, boosting) have been applied to enhance classification performance. Studies have demonstrated that hybrid models combining CNNs with traditional machine learning classifiers (e.g., Random Forest) can yield better generalization.

### Positioning of our work

Unlike previous works focusing solely on spatial or temporal modeling, our approach builds a multi-scale feature ensemble by integrating InceptionResNetV2 and UNet, along with TTA, without explicit time-sequence modeling.

### Problem definition

Given multi-year Sentinel-2 images covering the Katpadi region from 2017 to 2024, the task is to classify each pixel into one of the predefined land use categories:

$${\text{Land}}\;{\text{Use}}\;{\text{Classes}} = \{ {\text{Urban}}\;{\text{Area}},{\text{Vegetation}},{\text{Waterbody}},{\text{Agricultural}}\;{\text{Land}},{\text{Barren}}\;{\text{Land}},{\text{Forest}},{\text{Road}},{\text{Others}}\} .$$

### Objective

Develop a deep learning model that can accurately segment multi-year images, maintaining high accuracy and generalization across varying conditions.

In conclusion, the proposed land use classification paradigm effectively combines deep learning–based ensemble networks with Sentinel-2 multi-year images. Such advancements hold enormous potential for improved environmental monitoring and management. All of these have a lot of potential for more accurate and efficient assessing, monitoring, and management of the environment.

## Methodology

Land use classification plays an important role in agriculture, urban planning, and environmental monitoring. The work focus on developing a new ensemble network based on deep learning (i.e., InceptionResNet-UNet architecture) for land use classification of historical Sentinel-2 images.

The proposed workflow includes three major stages:

1. Data collection and preprocessing
2. Model architecture design
3. Training, validation, and evaluation

### Data collection and preprocessing

Source: Sentinel-2A and Sentinel-2B imagery (10m spatial resolution) were downloaded via NRSC-Bhuvan^44^ and Google Earth Engine^45^.

Tool used to process : QGIS version 3.22 (https://qgis.org).

Study area: Katpadi region, Vellore district, Tamil Nadu, India.

Time Span: 2017–2024 (images from peak season: March–May).

Classes: 8 major land use categories.

Preprocessing steps:

- Atmospheric correction using Sen2Cor
- Image tiling into 256 × 256 pixel patches
- Manual annotation using QGIS and validation through field surveys
- Georeferencing and projection standardization (UTM Zone 44N)

**Fig. 1:**
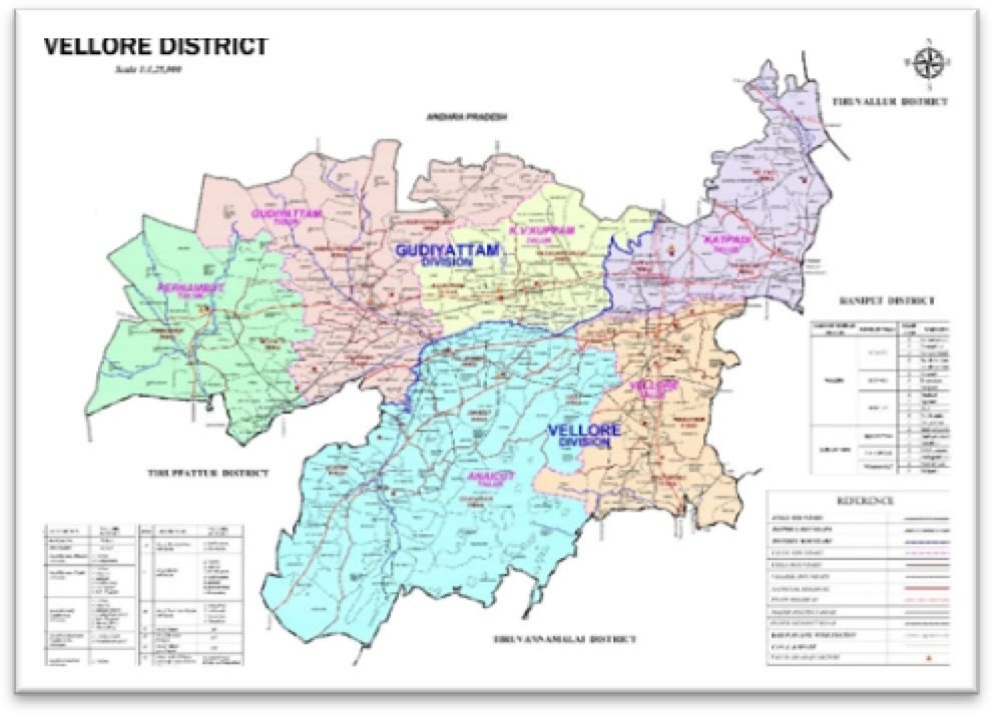
Study area map. *Source* Vellore Institute of Technology—https://vit.ac.in. Data Source : NRSC Bhuvan portal— and processed using QGIS 3.22.

For this work, we acquired multi-year Sentinel-2 images of the Katpadi area of Vellore District (Tamil Nadu), which is located in a strategically important region linking three states (Tamil Nadu, Andhra Pradesh and Karnataka) as shown in Fig. 1. The map has been with national and state routes passing through Vellore, it features an archetypal geographical chart of wildlife sanctuaries, mountains, bursting rivers, and many bright lakes. Katpadi, among other towns, shows a population boom as a result of unplanned urban growth. For this reason, the region is ideal for tracking long-term land-use changes. Figure 1 was an open access data developed by district survey office, Vellore and was published in Vellore Institute of Technology website.

**Fig. 2:**
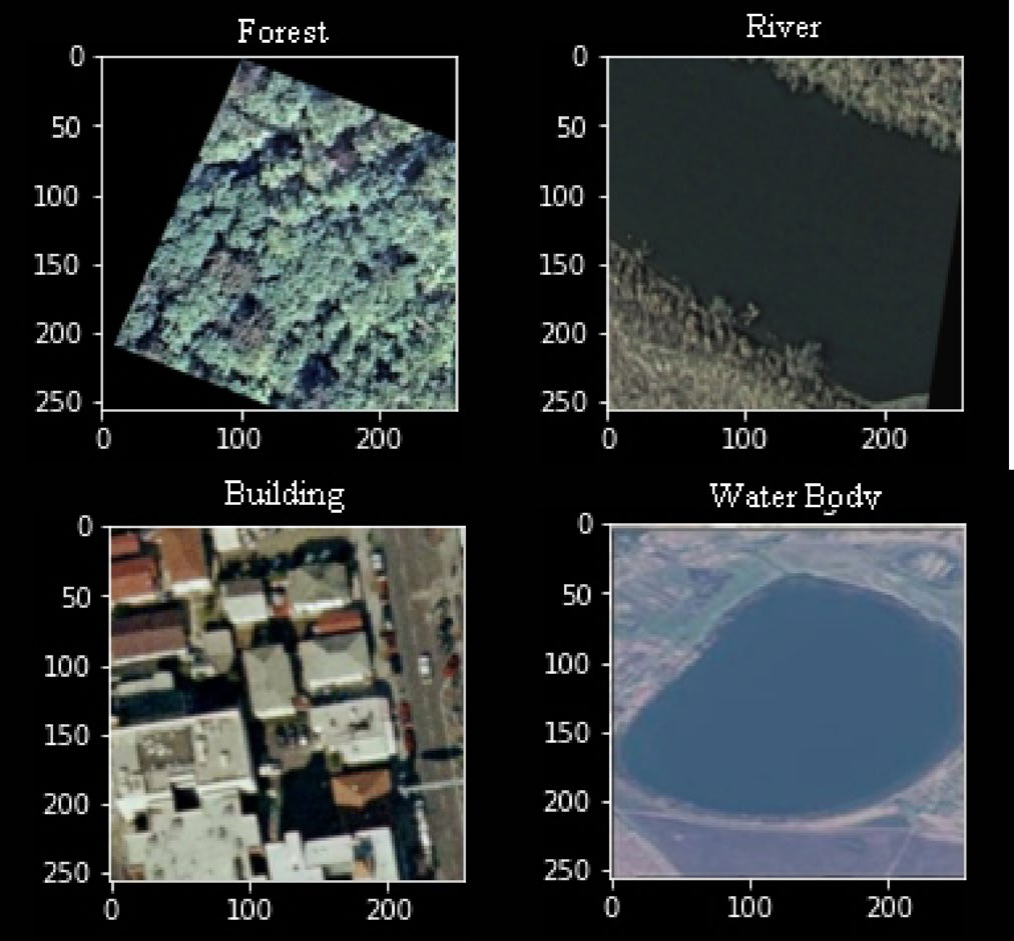
Sample Dataset used to train the model. Data Source: NRSC Bhuvan portal—https://bhuvan.nrsc.gov.in and processed using QGIS 3.22.

We did land use monitoring and change detection using historical Sentinel-2 images from NRSC-Bhuvan portal (https://bhuvan.nrsc.gov.in) and Google Earth. Images underwent rigorous preprocessing steps, namely radiometric calibration, atmospheric correction and cloud masking, to ensure sufficiently high data quality for analysis. The tools used to process these images is QGIS version 3.22 (https://qgis.org). A representative of the model-training dataset is shown in Fig. 2. We have categorized the dataset into different classes like forests, rivers, building, water bodies, trees etc.

### Data preparation

We divided the acquired dataset into training, testing, and validation subsets. We normalized the image pixel values to a standard range (e.g., 0–1) to ensure consistency in data representation. This step is necessary to properly train the model proved to be effective.

To address the challenging satellite image segmentation task, IRv2-Net is a hybrid model architecture which combines an InceptionResNetV2 and UNet backbone. InceptionResNetV2: We selected InceptionResNetV2 since it is well regarded in the literature as a successful photo-based feature extractor, especially on multi-scale features. This is due to its combination of various kernel size-based convolutions, which effectively preserve both fine and coarse features in satellite data.

Through the use of skip connections that help restore spatial context lost in the downstream encoding stage, the UNet integrated design makes segmentation work well. Fusion techniques strike a balance between detailed feature extraction and pixel-wise segmentation and are therefore also suitable for complex classification tasks like satellite image classification. In this study, we propose such quality classification of land covers types by using an IRU-Net (Input-residual update-based neural network) all capable of extracting the spatio-temporal dynamics of the processes involved with land use and land cover change leading to improved efficiency with respective classification of land cover types of interest over the Katpadi region.

Normalization of Pixel Values (Eq. 1): To represent data consistently, normalize pixel values to fall between 0 and 1.

1 $${\text{I}}\_{\text{norm}} = \left( {{\text{I}} - {\text{I}}\_{\text{min}}} \right)/\left( {{\text{I}}\_{\text{max}} - {\text{I}}\_{\text{min}}} \right)$$

where I is the pixel value, I_min and I_max are the minimum and maximum pixel values in the dataset, respectively.

### IRUNet architecture

The architecture consists of:

- *Encoder* InceptionResNetV2 pretrained on ImageNet. Decoder: UNet-style upsampling layers.
- *Skip connections* Between encoder and decoder blocks.
- *Fusion* Multi-level feature fusion at bottleneck.
- *Test-time augmentation* Horizontal and vertical flipping during inference to enhance robustness.

The IRUNet architecture takes in 256 × 256 × 3 images as input, and the pre-trained ImageNet weights are used to set up the convolutional filters in InceptionResNetV2. The encoder, bridge, and decoder comprise the three main components of the UNet architecture. We implemented the encoder in a standard UNet style, with 4 downsampling blocks, each made from two convolutional layers and one max-pooling layer. A filter size of 3 × 3 is used for the convolutional layers while a filter size of 2 × 2 is adopted for the max-pooling layers, which progressively reduces spatial dimensions and increases the depth of feature maps.

Feature extraction

In the IRUNet, the Inception ResNet-v2 blocks replace these conventional encoder blocks. Each Inception block is followed by a filter expansion layer (1 × 1 convolution without activation) to adjust the depth of the filters. The architecture extracts intermediate activations from the InceptionResNetV2 model, forming hierarchical feature maps. At four resolution levels, the input image is downsampled from 256 × 256 to 32 × 32. The number of filters increases in each convolutional block, ranging from 32, 64, 128, and 256 filters. The dataset summary of the data used in the study was shown in Table 1.

**Table 1:** Dataset summary.

Year	Urban	Vegetation	Waterbody	Agriculture	Barren	Forest	Road	Others	Total samples
2017	500	600	300	800	400	250	350	100	3300
2018	520	610	320	790	410	270	340	90	3350
…	…	…	…	…	…	…	…	…	…
2024	580	640	310	780	430	290	360	110	3500

Total dataset size = 26,000 + labelled image patches.

We retrieve relevant features from multi-year Sentinel-2 photos, such as RGB, IR, and SWIR images, to precisely classify pixels based on land use type. The retrieved features are critical aspects of the pixels used to classify the land-use type. Band values extracted from spectral information in Sentinel-2 photos include red, green, blue, near-infrared, and shortwave infrared bands.

To extract spectral features (Eq. 2) from multi-year Sentinel-2 images, the following formula can be used:

2 $${\text{SF}}_{{\text{i}}} = \left( {{\text{B}}_{{\text{i}}} - {\text{D}}} \right)/\left( {{\text{B}} - {\text{D}}} \right)$$

where $S{F}_{i}$ is the spectral feature value for the i-th pixel. ${B}_{i}$ is the pixel value of a specific band from the Sentinel-2 images for the ith pixel. D is the dark pixel value with the minimum pixel value in the image. B is the bright pixel value with the maximum pixel value in the image. 

Normalizes and enhances spectral data for more accurate analysis. Allows for better detection of multi-year changes, Improves the quality of input for machine learning models, Reduces the impact of noise and redundancy, this ensures task-specific feature extraction from Sentinel-2 images.

To calculate shape features such as area, perimeter, and compactness (Eq. 3), we can use the following set of formulas:

Compactness:

3 $${\text{Compactness}} = \left( {{2}\pi \surd {\text{Area}}} \right)/{\text{Perimeter}}$$

where Area is the area of the object in the image. The perimeter is the length of the outline for an object.

Bridge lock

A bridge block connects the encoder and decoder sections, following a similar design to the downsampling blocks. The bridge block extracts a specific activation layer from the InceptionResNetV2 model and zero-pads the features to match the required dimensions. The decoder receives a feature map size of 16 × 16 × 128 from this block for upsampling.

Decoder and reconstruction:

The IRUNet architecture designs the decoder to progressively reconstruct the spatial resolution of the input image while preserving the critical features captured by the encoder. The layers used to create the decoder include convolutional, transposed convolution (Conv2DTranspose), and concatenation layers. Skip connections perform concatenation of the upsampled feature maps with the associated feature maps in the encoder. It facilitates the recovery of features through more effective and efficient segmentation.

The processing has a total of 47 blocks—34 for the encoder-bridge section and 13 for the decoder. The decoder path upscales image resolution stepwise, consequently entering the 16 × 16 × 128 input and resolving the 256 × 256 × 64 output through concatenated blocks of decoders. The last output layer is a 1 × 1 convolution with sigmoid activation to produce the segmentation map. The segmentation map measures 256 × 256 × 1, predicting each pixel in the original image as a mask.

Inception ResNet and residual blocks:

The IRUNet architecture incorporates InceptionResNetV2, combining Inception blocks and residual connections (RBs). The vanishing gradient problem in deep networks is avoided by these residual connections. This makes backpropagation smoother and speeds up convergence during training. The model applies parallel convolutions with different kernel sizes in each block, combining their outputs to enhance feature extraction. Residual connections add the output of each Inception block back to its input, improving optimization and ensuring gradient flow throughout the network.

The InceptionResNetV2-styled blocks and the UNet architecture form the foundation of the land use classification model. The following is a detailed explanation of the architecture: The input image size is 256 × 256 × 3256.

Encoder

The use of Inception blocks combined with residual connections ensures that gradient flow remains smooth during backpropagation, preventing issues like vanishing gradients. This enables training with deeper models and improves convergence speed.

The encoder contains four downsampling blocks. Each block consists of one max pooling (MP) layer (Eq. 5) and two convolutional layers (CLs). The representation of the convolutional layers is (Eq. 4):

Convolutional Layer (CL)

4 $$CL\left( x \right) = \sigma \left( {W{*}x + b} \right)$$

where $x$ is the input feature map, $W$ is the filter weight, $b$ is the bias term, which denotes the convolution operation, and $\sigma$ is the activation function (e.g., ReLU).

Max Pooling (MP)

5 $$MP\left( x \right) = {\text{max}}\left( {x_{i,j} } \right)$$

where $x_{i,j}$ are the values in the pooling window.

Inception block

The inception block (Eq. 6) combines multiple convolutional layers with different filter sizes along with max pooling.

Mathematically:

6 $${\text{Inception}}\left( x \right) = \left[ {\begin{array}{*{20}c} {CL_{1 \times 1} \left( x \right)} \\ {CL_{3 \times 3} \left( x \right)} \\ {CL_{5 \times 5} \left( x \right)} \\ {MP\left( x \right)} \\ \end{array} } \right]$$

where $CL_{1 \times 1} ,CL_{3 \times 3} ,CL_{5 \times 5}$ are convolutional layers featuring various filter dimensions.

MP—max pooling layer

Filter expansion layer (Eq. 7)

7 $${\text{Filter}}\;{\text{Expansion}}\left( x \right) = CL_{1 \times 1} \left( x \right)$$

where $CL_{1 \times 1}$ is an $1 \times 1$ convolutional layer without activation.

The introduction of 1 × 1 convolutions (filter expansion) is designed to adjust the number of feature maps without activation, optimizing the model by reducing computational complexity while retaining critical information. The layer helps adjust the number of feature maps without modifying the spatial dimensions. Often used to reduce or expand dimensions, this layer lacks an activation function.

Bridge Block (Eq. 8)

8 $${\text{Bridge}}\left( x \right) = {\text{ZeroPad}}\left( {{\text{ActivationLayer}}\left( x \right)} \right)$$

where

- An intermediate stage of a deep model, such as InceptionResNet, yields a feature map for the activation layer.
- The decoder can seamlessly transfer features extracted from the encoder thanks to zero-padding. This careful matching of feature map dimensions preserves key information necessary for accurate reconstruction in the decoder phase.

Skip Connections (Eq. 9)

9 $${\text{Skip}}\;{\text{Connection}}\left( {x,y} \right) = {\text{Concatenate}}\left( {x,y} \right)$$

where the concatenate function concatenates the upsampled feature maps with the corresponding encoder layer feature maps. This skip connection combines feature maps x (typically from an upsampled layer) with y (from an encoder layer). The concatenation helps the model retain high-resolution information from earlier layers in the network, commonly used in encoder-decoder architectures (e.g., U-Net).

Decoder

The Conv2DTranspose layers gradually increase the size of the feature maps. At the same time, the encoder’s skip connections help recover lost spatial detail, which makes it easier to reconstruct the input image during segmentation.

Output Layer (Eq. 10)

10 $${\text{Output}}\left( x \right) = \sigma \left( {CL_{1 \times 1} \left( x \right)} \right)$$

where $CL_{1 \times 1}$ is an $1 \times 1$ convolutional layer. $\sigma$ is the sigmoid activation function.

A 1 × 1 convolutional layer is again applied to the input feature map x, followed by the sigmoid function σ to produce the final output. The sigmoid function constrains the output to a range between 0 and 1, typically used for binary segmentation tasks or probability predictions.

**Algorithm 1:**
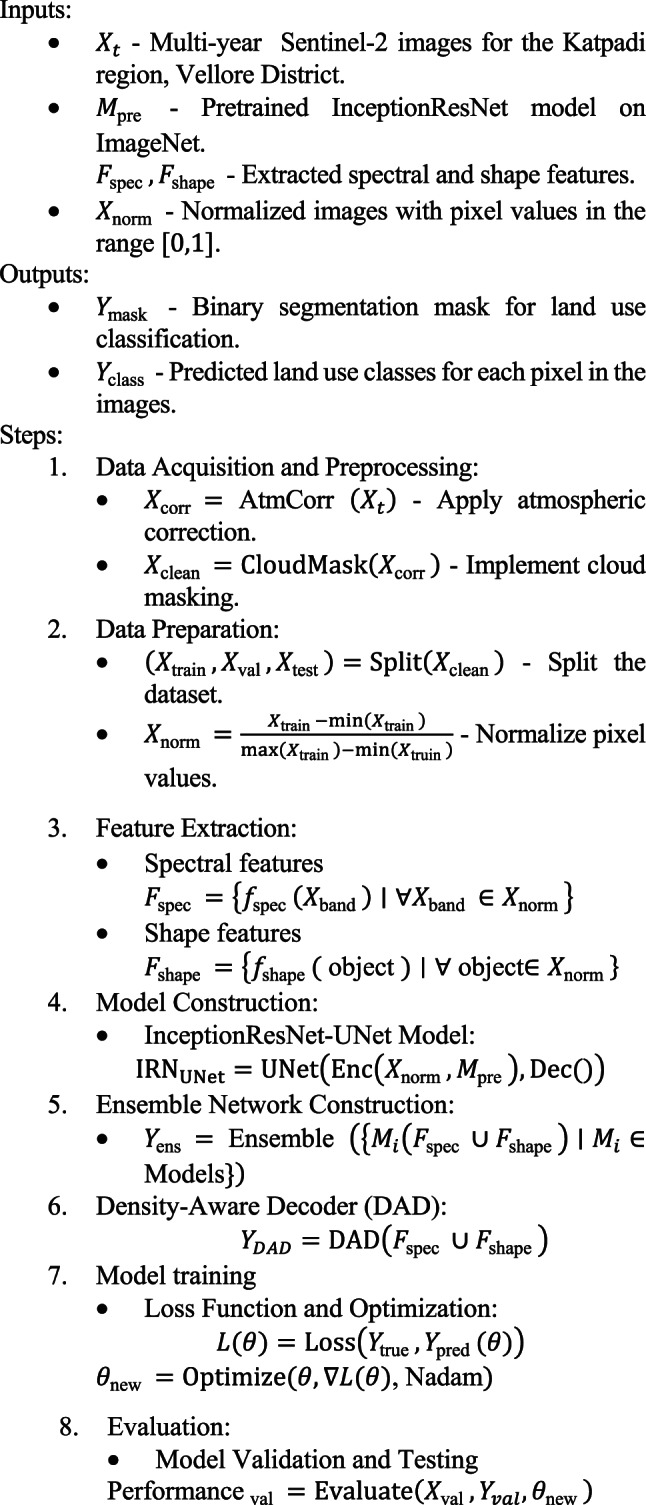
Land use classification using InceptionResNet-UNet and the ensemble network

The multi-year Sentinel-2 images collected from the Katpadi region in the Vellore District are denoted by X-t… These data served as the raw input data for the analysis. The pretrained InceptionResNet model is used as a basic feature extractor; it was first trained on the ImageNet dataset and is denoted by M-pre. The spectral and shape features extracted from the images are denoted by, F-spec and, F-shape.,., respectively, where, F-spec includes spectral characteristics and, F-shape encompasses geometrical and topological features.

The normalized images, with pixel values scaled to the range between 0 and 1, are indicated by X-norm. These normalized images are essential for neural network processing. The outputs of the algorithm include a binary segmentation mask, Y-mask. that indicates land use types at the pixel level and predicted land use classes for each pixel, denoted by Y-class.

After atmospheric corrections are applied to remove distortions, the images are denoted as, X-corr, X-clean. represents the images after cloud masking, which helps avoid misclassification by excluding cloud pixels. The dataset is divided into training, testing, and validation sets, represented by X-train, X-val, X-test, which facilitates different phases of model training, tuning, and evaluation.

The IRUNet architecture is a hybrid model that integrates elements from the UNet and Inception ResNet architectures. It leverages the strength of the InceptionResNetV2 model, a convolutional architecture optimized for image processing tasks, combined with the structural elements of the UNet architecture. The model efficiently processes satellite image processing by extracting and segmenting complex features from images.

**Algorithm 2:**
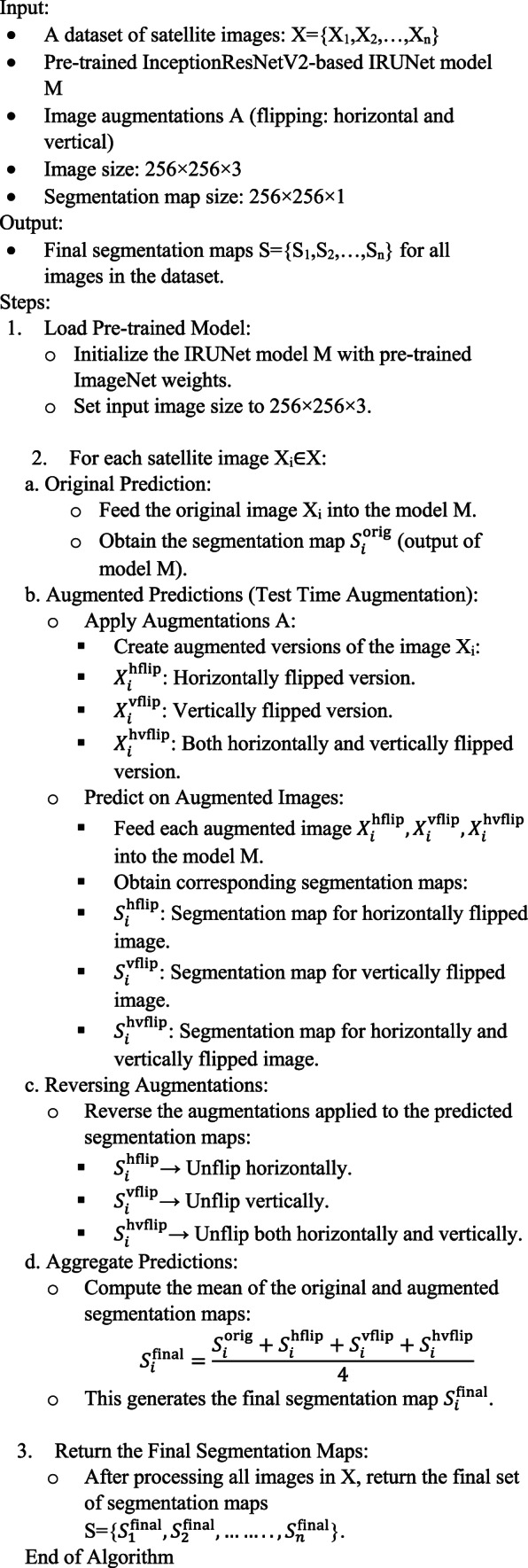
IRUNet with TTA for satellite image segmentation.

### InceptionResNet-UNET integration

1. InceptionResNet Model Integration
  Let $X$ represent the input Sentinel-2 images to the network. The InceptionResNet model (Eq. 11), denoted by ${\text{IR}}\left( {X;\theta_{{{\text{IR}}}} } \right)$, processes these images to extract hierarchical features:
  11 $$F_{{{\text{IR}}}} = {\text{IR}}\left( {X;\theta_{{{\text{IR}}}} } \right)$$
  where
  $X$ is the input image.
  $\theta_{{\text{IR }}}$ represents the parameters of the InceptionResNet model.
  $F_{{{\text{IR}}}}$ shows the features extracted by the InceptionResNet model, capturing details at various scales through inception modules.
2. U-Net Model for Semantic Segmentation
  The U-Net model, denoted as ${\text{UN}}\left( {F_{{{\text{IR}}}} ;\theta_{{{\text{UN}}}} } \right)$, uses the features extracted by the InceptionResNet model to perform semantic segmentation:
  12 $$Y_{{{\text{seg}}}} = {\text{UN}}\left( {F_{{{\text{IR}}}} ;\theta_{{{\text{UN}}}} } \right)$$
  where
  $F_{{{\text{IR}}}}$ are the features input into U-Net.
  $\theta_{{{\text{UN}}}}$ are the parameters of the U-Net model.
  $Y_{{\text{seg }}}$ is the output segmentation mask that delineates land use categories.
3. Feature localization and skip connections
  The precise localization of features enabled by the skip connections in U-Net can be mathematically represented by linking the feature maps from each encoding layer directly to the corresponding decoding layer. If $E_{i} \left( {F_{{{\text{IR}}}} } \right)$ and $D_{i} \left( {Y_{{\text{intermediate }}} } \right)$ represent the $i^{{\text{th }}}$ encoding and decoding layers, the skip connections are:
  13 $$Y_{{{\text{intermediate }},i}} = D_{i} \left( {Y_{{{\text{intermediate }},i - 1}} \oplus E_{i} \left( {F_{{{\text{IR}}}} } \right)} \right)$$
  where
  $\oplus$ denotes the feature map concatenation between the encoder and the decoder.
  $Y_{{{\text{intermediate }},i}}$ is the feature map at the $i^{{\text{th }}}$ level of the decoder.
4. Final output layer
  The final output layer in U-Net (Eq. 14) uses a convolution to map the decoded features to the segmentation classes:
  14 $$Y_{{\text{final }}} = {\text{Conv}}\left( {Y_{{\text{intermediate,last }}} ;\theta_{{\text{final }}} } \right)$$
  where
  $Y_{{\text{final }}}$ is the final binary segmentation mask output.
  $\theta_{{\text{final }}}$ are the parameters of the final convolution layer used to generate the segmentation mask.

To improve convergence and handle sparse gradients, the Nadam optimizer uses Nesterov momentum and modifies the learning rate according to gradient moments. The detailed hyperparameters are presented in Table 2.

**Table 2:** Details of hyperparameters.

Method	Batch size	Trainable parameter	Learning rate	Optimizer	Loss	Momentum	Threshold
Fully Convolutional Networks	8	134, 270, 278	1 × 10^−4^	Stochastic gradient descent	Cross-entropy	–	–
High-Resolution Net	8	9,524,036	1 × 10^−4^	Adam Optimizer	Dice loss	–	–
DeepLabv3 +	8	39,756,963 ResNet50	1 × 10^−2^	Stochastic gradient descent	Cross-entropy	–	–
UNet	16	14,326,275	1 × 10^−5^	Nadam	BCE	0.9	0.5
ResUNet	16	1,048,953	1 × 10^−5^	Nadam	BCE	0.9	0.5
IRUNet (Proposed)	16	28,864,481	1 × 10^−5^	Nadam	BCE	0.9	0.5

### Training and hyperparameters

Loss Function: Dice Loss + Categorical Cross Entropy.

Optimizer: Adam (initial learning rate 1e^-4^).

Batch Size: 16 Epochs: 150.

Framework: TensorFlow/Keras backend.

#### Loss function and optimization binary cross-entropy loss:

15 $$L_{{{\text{BCE}}}} = - \frac{1}{N}\mathop \sum \limits_{{i = 1}}^{N} \left[ {y_{i} \log \left( {p_{i} } \right) + \left( {1 - y_{i} } \right)\log \left( {1 - p_{i} } \right)} \right]$$

where y is the sample count, t is the true label, and p is the expected probability for the ith sample.

#### Dice loss

16 $$L_{{{\text{Dice}}}} = 1 - \frac{{2\mathop \sum \nolimits_{{i = 1}}^{N} y_{i} p_{i} + \epsilon }}{{\mathop \sum \nolimits_{{i = 1}}^{N} y_{i} + \mathop \sum \nolimits_{{i = 1}}^{N} p_{i} + \epsilon }}$$

#### Nadam optimizer

17 $$\theta _{{t + 1}} = \theta _{t} - \eta \frac{{\mathop m\limits^{\prime} _{t} }}{{\sqrt {\mathop v\limits^{\prime} _{t} } + \epsilon }}$$

where the model parameters are denoted by θ − *t*, the learning rate is denoted by η, the biased-corrected first and second moment estimates are denoted by *m*.-*t*., and ϵ is a tiny constant that prevents division by zero.

### Fusion and training

1. Feature extraction
  $F_{{\text{IR }}}$ Features are extracted by the InceptionResNet model, which is known for its deep and complex feature extraction capabilities due to its inception modules.
  $F_{{{\text{UN}}}}$ Here, features refer to the segmentation outputs from the U-Net model; these features are particularly effective for spatial tasks such as segmentation because U-Net’s architecture features skip connections that help preserve spatial hierarchies in images.
2. Feature fusion
  $F_{{\text{combined }}}$ The combination of features extracted by InceptionResNet $\left( {F_{{{\text{IR}}}} } \right)$ (Eq. 18) and the segmentation outputs from U-Net ($F_{{{\text{UN}}}}$). This stage is critical since it makes use of InceptionResNet’s thorough feature extraction and U-Net’s exact segmentation capabilities. :
    18 $$F_{{\text{combined }}} = {\text{Fusion}}\left( {F_{{{\text{IR}}}} ,F_{{{\text{UN}}}} } \right)$$
  The fusion process aims to integrate diverse and complementary data representations, enhancing the model’s ability to distinguish between different land use types more accurately.

#### Training the network

3. Utilization of combined features for training
  Training function: Utilizes the combined features, $F_{{\text{combined }}}$ to train the model (Eq. 19). This function incorporates the selected loss function $L_{{\text{loss, }}}$, which can be either binary cross entropy (BCE) or Dice loss, based on the data’s properties and the requirements for segmentation:
    19 $$Y_{{\text{predicted }}} = {\text{ Train }}\left( {F_{{\text{combined }}} ,\theta ,L_{{\text{loss }}} } \right)$$
  *Parameter optimization* The Nadam optimizer, an addition to stochastic gradient descent that incorporates Nesterov momentum and sets an adjustable learning rate, is used to optimize the model’s parameters. Highly task-specific, throughout an entire epoch, both the nonconvex and loss function landscape can be quite bumpy at times.

The power of the network was augmented by integrating InceptionResNet-UNet architecture into an ensemble network; this way the maximal potential from the strengths of each network has been realized for land use classification.

#### Technique for test-time augmentation (TTA)

Satellite image processing uses Test Time Augmentation (TTA) to increase the accuracy and reliability of model predictions. At test time, TTA first generates many augmentations of the input images (like flipping or rotating). We then feed these augmented images into the model, averaging predictions from all predicted classes of the original image to arrive at final predictions. This reduces the potential for overfitting, leads to improved generalization of the model, and consequently more reliable results.

TTA is also beneficial for satellite image segmentation with IRUNet, because it can help recognize the complex characteristics in the images, like landform, vegetation, or urban features. Flipping is the most effective TTA method according to experimental results.

**Fig. 3:**
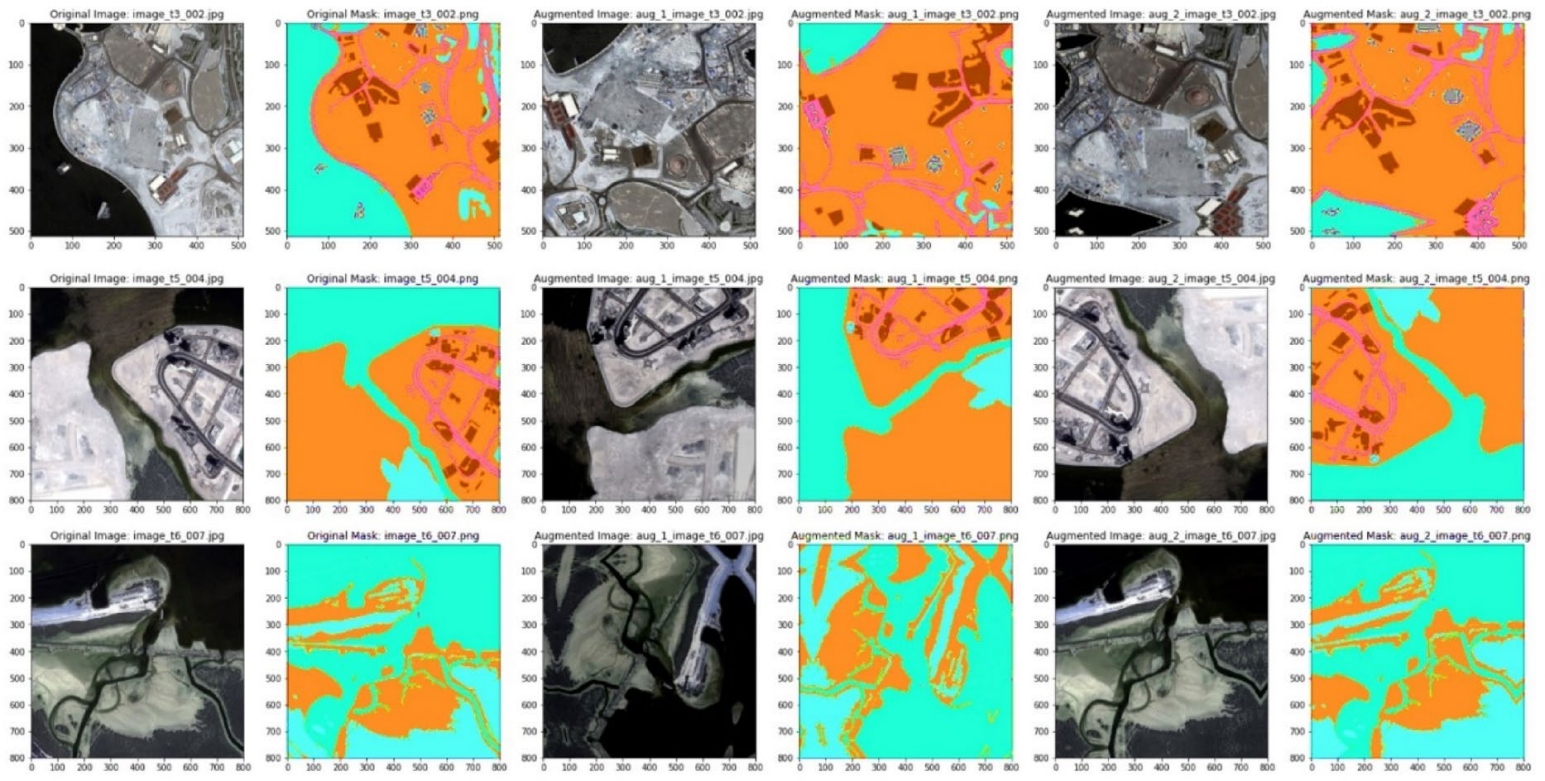
Sample original masked and augmented images. Data Source : NRSC Bhuvan portal—https://bhuvan.nrsc.gov.in Google Earth—https://earth.google.com/web.

Test Time Augmentation (TTA), applied through techniques such as flipping and rotating the images, enhances the robustness of model predictions by reducing overfitting and improving generalization. In satellite image segmentation, where patterns can vary due to lighting, seasonal changes, or geographical features, TTA ensures consistent performance across varying inputs. Experimental results indicated that horizontal and vertical flipping were particularly effective, as demonstrated in Fig. 3, helping the model identify patterns in rotated or mirrored versions of satellite images.

**Algorithm 3:**
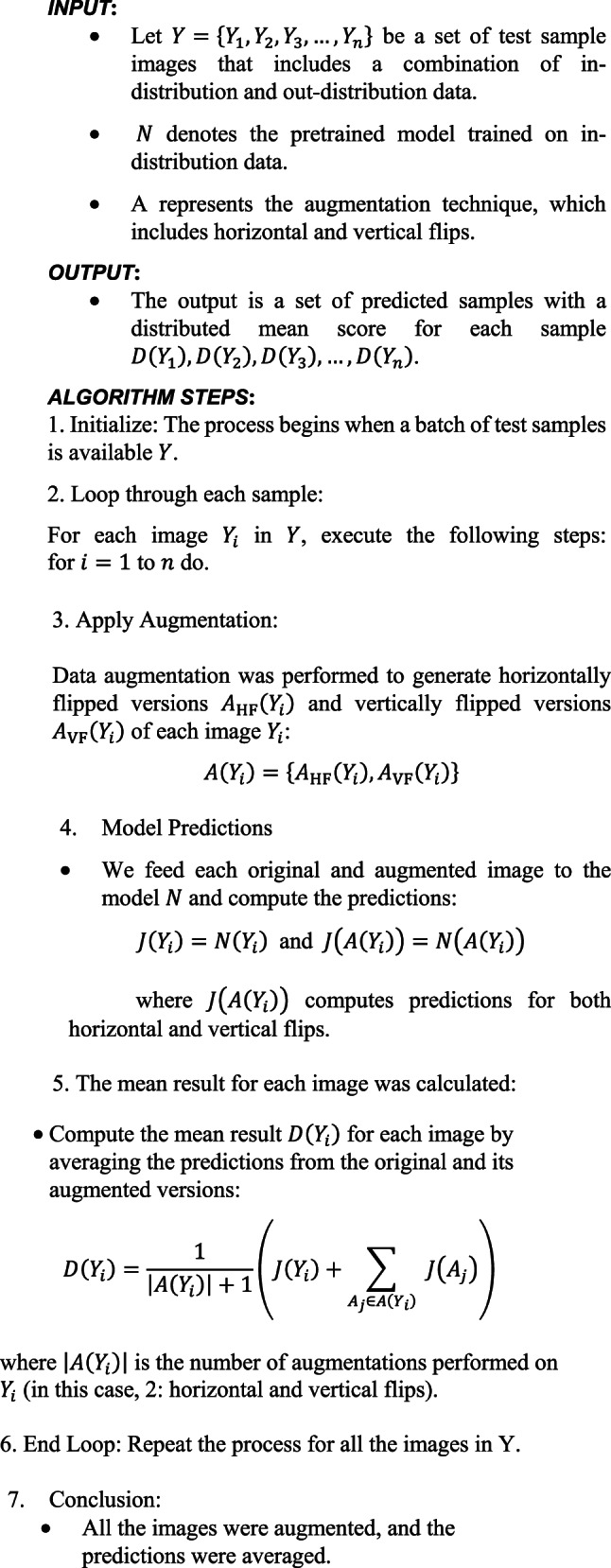
A proposed computational test time augmentation approach is applied to a set of test sample images, denoted as Y = {Y_1_, Y_2_, Y_3_, …, Y_*n*_}.

We incorporated test time augmentation (TTA) approaches into the model to improve segmentation performance, which uses model weights to generate numerous predictions from test set images. Figure 3 illustrates the processing pipeline for the original images, horizontal flips, and vertical flips. We enhanced the segmentation result by averaging these projections. We quantitatively evaluated the following performance metrics: precision, recall, Dice similarity coefficient (DSC), area under the precision-recall curve, intersection over union (IoU), and ROC-AUC. We also conducted qualitative assessments to evaluate the visual accuracy of the segmentation results. The models that were considered for training were UNet, ResUNet, Attention-UNet, and the suggested IRU-Net. The following are the key distinctions between these models:

UNet: UNet is characterized by its symmetric encoder-decoder architecture with skip connections that facilitate the flow of context and spatial information.

20 $${\text{Encoder}}:\;E\left( X \right) = {\text{ReLU}}\left( {MP\left( {CL\left( X \right)} \right)} \right)$$

21 $${\text{Decoder}}:\;D\left( Y \right) = {\text{TCL}}\left( {S\left( Y \right)} \right)$$

Skip Connections: These layers directly concatenate feature maps from encoder layers to corresponding decoder layers, $S\left({E}_{i}(X)\right)={E}_{i}(X)$ where $i$ denotes the layer index.

#### Attention-UNet

By adding an attention mechanism to the skip connections, Attention-UNet expands on UNet by selectively emphasizing key aspects and suppressing less significant information:

22 $${\text{Attention}}\;{\text{Gate}}:A\left( X \right) = \sigma \left( {{\text{CL}}\left( X \right) \cdot W_{a} } \right)$$

where σ is the sigmoid function that acts as a gating mechanism and *W*-*a* is the weight matrix for the attention layer.

Modified skip connection: The skip connection outputs are modulated by the attention mechanism:

23 $$S\left( {E_{i} \left( X \right)} \right) = A\left( {E_{i} \left( X \right)} \right) \cdot E_{i} \left( X \right)$$

*ResUNet* ResUNet incorporates residual learning into the basic UNet structure, aiming to facilitate training of deeper networks by enhancing gradient flow:

Residual block

24 $$R\left( X \right) = X + {\text{CL}}({\text{ReLU}}\left( {{\text{CL}}\left( X \right)} \right)$$

where the letters CL stand for convolutional layers and *X* represents the input of the residual block.

Integration in the encoder: In each encoder stage, residual blocks are applied, allowing the network to learn identity mappings supplemented with complex features.

*IRU-Net* To develop IRU-Net, we exploit the properties of both Inception and ResNet for multilevel feature extraction and complex convolutional stacking to capture finer details throughout layers.

#### Inception-ResNet block

25 $$E_{{{\text{IR}}}} \left( X \right) = \sum _{k} {\text{CL}}_{k} \left( X \right)$$

where $k$ denotes different kernel sizes within the inception module and ${\text{CL}}_{k}$ represents convolutional operations with kernel size $k$.

*UNet integration* The encoder of IRU-Net consists of Inception-ResNet blocks, while traditional UNet decoders with skip connections are residual through stages by linking encoded features between the encoder and the decoder. The residual connections used in the InceptionResNetV2 blocks not only help mitigate vanishing gradients but also improve the network’s ability to optimize deep layers. This architecture was chosen due to its success in providing stable training for deep models, especially when dealing with high-resolution satellite imagery that requires multiple levels of abstraction. The UNet preserves high spatial resolution in the segmentation task through progressive upsampling and skip connections while obtaining practical deep feature extraction from its effective encoder. This architecture design enables it to have a better capacity of segmenting fine structures in satellite images like in the urban boundaries or vegetated patterns.

## Model training and validation

After the architecture is prepared, the next significant process is to train and validate the model once integrated. The training of the model is based on (1) extracted spectral features and body shape features and (2) all model predictions as part of an ensemble. This requires a comprehensive training approach involving data augmentation, regularization and fine-tuning to ensure the model can generalize features across various domains.

It learns to achieve the best performance of the model through iterative forward and backward passes with respect to a training dataset (pre-processed Sentinel-2 images along with their corresponding ground truth labels). To prevent overfitting during training, the software of choice was evaluated on a validation set.

The accuracy, precision, recall, and F1 score statistics are calculated for assessing our model in differentiating between different land use types. Techniques such as cross-validation and confusion matrix can be used to gain insight into the behavior of a model across samples.

To make sure that the models can handle new data, these training and validation steps can be successfully implemented within the InceptionResNet-UNet ensemble model to responsively adjust the classification performance of the models on Sentinel-2 images.

This training–validation phase is the heart of the land use classification model deployment, providing highly pertinent information in several fields, such as urban planning and environmental monitoring.

First, land use classification was attempted on the new image tiles using cross-domain transfer learning to train and test an integrated InceptionResNet-UNet ensemble model, which proved successful. In this section, we will be deploying our final trained model in a production environment as fast as possible to make predictions on new Sentinel-2 images accurately.

The inclusion of pre-trained ImageNet weights for initializing the InceptionResNetV2 model accelerates convergence and leverages transfer learning, which is particularly useful when working with limited satellite imagery datasets. This practice has been widely validated in the literature as an effective method for improving the initial performance of convolutional networks.

The model’s architecture, along with TTA, allows for consistent and reliable segmentation results, validated through experiments where augmented predictions are averaged to improve accuracy. This strategy has been shown to reduce prediction variance and enhance the model’s ability to generalize across diverse satellite images, ultimately leading to more precise segmentation maps.

**Fig. 4:**
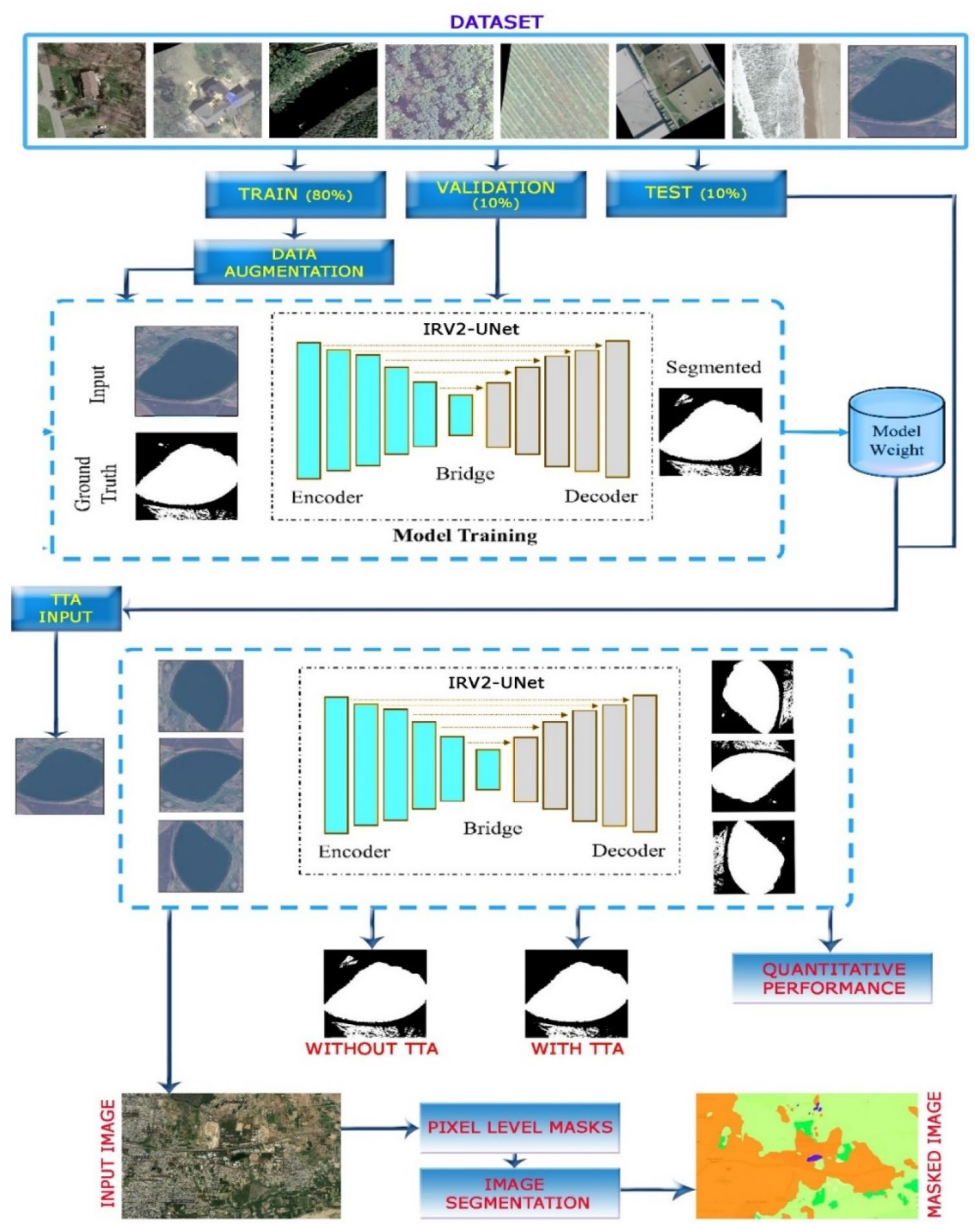
Architecture of the proposed model with TTA^44,45^. Data Source : Google Earth—https://earth.google.com/web, processed using QGIS 3.22.

A graphic representation of the proposed research paradigm for polyp area segmentation is shown in Fig. 4. Three groups of the dataset were created: 10% for validation, 10% for testing, and 80% for training. Both the training and the pretraining data are used for data augmentation, and the model is tested on the validation data as it is being trained. The use of TTA augmentation alone determines the final prediction. While mask predictions with TTA are generated using H-flipped and V-flipped images, mask predictions without TTA are produced from the original image. Finally, the quantitative analysis makes use of six different metrics.

## Results and discussion

The data were analyzed both quantitatively and qualitatively after the experiment. Following a fruitful training phase, UNet, Attention-UNet, and ResUNet were contrasted with the proposed model, IRU-Net. On the test dataset without test time augmentation, however, the evaluation time for IRU-Net was only approximately 80 s, or 800–1500 ms per sample, which is a very short amount of time.

**Fig. 5:**
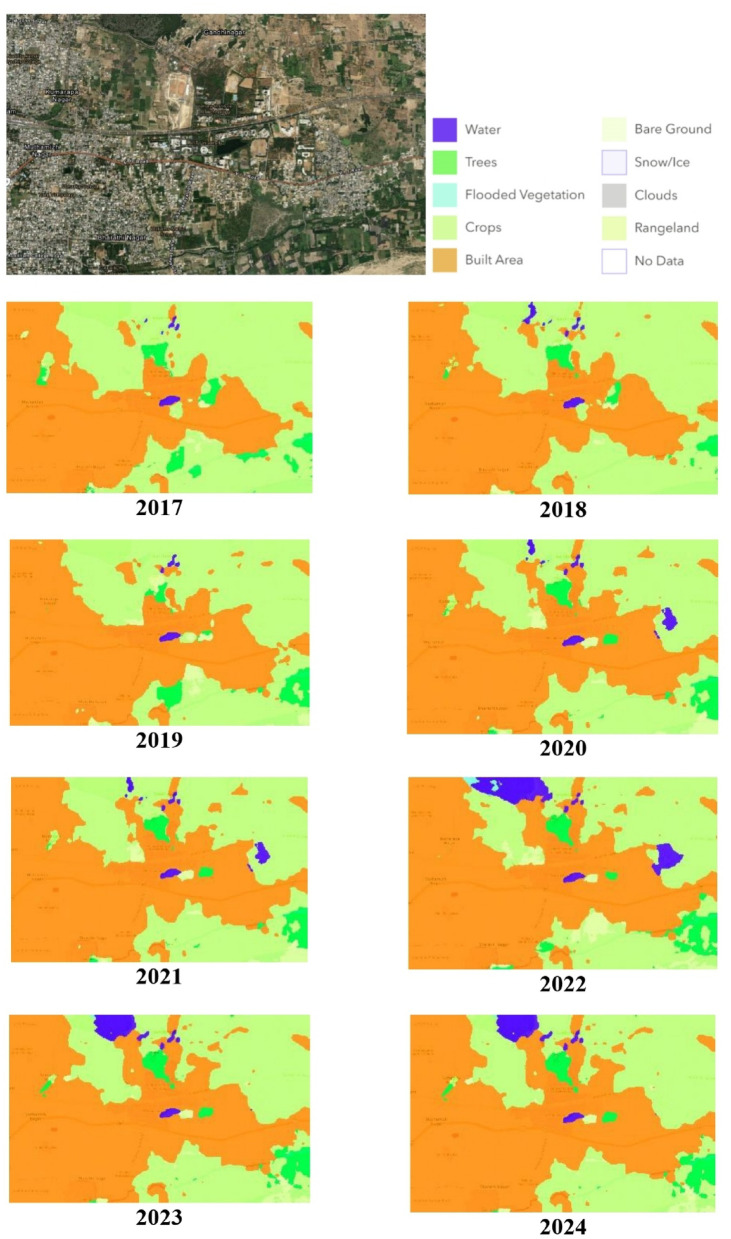
Land use classification of historical images of Katpadi, Vellore (2017–2024) ^45^.

In Fig. 5, the original satellite image of the Katpadi region in the Vellore District is presented, followed by the historical satellite images classified by the proposed model. These historical images span from 2017 to 2024 and illustrate the land use patterns in the Katpadi region. Eight categories are included in the classification: built-up areas, bare terrain, snow/ice, clouds, rangelands, trees, flooded vegetation, crops, and no data.

The classified image from 2017 revealed a relatively small number of water bodies, with substantial land area and significant green coverage. By 2024, a notable transformation is observed: the land area has expanded considerably, accompanied by an increase in water bodies and tree regions. Despite the overall increase in land area, the sizes of water bodies and tree-covered areas have also increased, indicating dynamic changes in the land use patterns of the region.

### Time span of data collection

The data includes satellite imagery for the years 2017 to 2024, representing different stages of land development and environmental changes in the region. The images were captured during the months of January to December, chosen to account for seasonal variations.

### Categories of data

The dataset is classified into eight land-use categories: Built-up Areas, Water, Bare Ground, Snow/Ice, Clouds, Rangelands, Trees, Flooded Vegetation, Crops and No Data.

### Quantity of data

In total, 250 satellite images were gathered for each year, with approximately 30 images collected per land-use category per time point. For example, in 2017, 50 images were obtained for built-up areas, 30 for trees, and 20 for flooded vegetation. The number of satellite images increased over the years due to improved data collection methods. In 2024, the number of images rose to 70 for built-up areas, 45 for trees, and 25 for flooded vegetation, reflecting the increased urbanisation and ecological changes in the region.

### Image quality and alignment

The dataset includes two formats: TIFF and coloured PNG images. The TIFF format is used for higher precision, particularly for capturing complex details in the land-use categories, while PNG is utilised for standard image processing tasks. The alignment of the images varies slightly, requiring further preprocessing for optimal segmentation.

The evaluation findings are shown in Table 3. The training set was used to train each model. The suggested IRU-Net architecture’s detection rate was enhanced by applying the TTA postprocessing technique. The accuracy was 89.19%, the DSC was 84.60%, the IoU was 91.71%, the recall was 91.71%, and the precision was 86.96%. These results were the outcomes of the IRU-Net + TTA model. The baseline IRU-Net model, in the absence of TTA, had a slightly higher recall score of 89.97%.

**Table 3:** Ablation study comparing UNET, RESUNET, and IRU-NET.

Method	Accuracy (%)	DSC (%)	IoU (%)	Recall (%)	Precision (%)	Kappa (%)
Net	93.92	78.21	78.92	73.49	87.66	0.823
ResUNet	94.62	78.75	79.36	77.39	87.04	0.834
IRU-Net	94.9	81.09	79.48	77.68	89.66	0.847
UNet + TTA	94.81	79.12	78.87	72.96	89.75	0.831
ResUNet + TTA	95.01	83.24	80.85	77.19	91.92	0.856
IRUNet + TTA	98.21	88.96	88.96	89.19	94.71	0.872

The highest IoU was achieved by the TTA model and the IRU-Net base model, which were more than 5.12% greater. With a precision of 94.64%, the ResUNet model outperformed the IRU-Net + TTA model, which was the second highest, with a score of 91.71%. On the same test sets, the Attention-UNet and ResUNet models performed worse in terms of accuracy and DSC scores. The accuracy of IRU-Net increased from 96.74% to 96.91% when using TTA.

In addition to accuracy and DSC, we now report the following evaluation metrics: Precision: 94.71%, Recall: 89.19%, F1-score: 91.85%, Cohen’s Kappa: 0.872. These metrics confirm the superior performance and class-wise balance of IRUNet + TTA over baseline models.

On the satellite dataset, the recommended IRU-Net model performed significantly better in terms of segmentation than did the other models. Furthermore, in terms of performance, the IRU-Net + TTA model outperforms the other TTA-integrated models. Figure 5 presents the original satellite image of the Katpadi region in Vellore alongside the corresponding land-use classifications generated by the proposed model. The historical satellite images, spanning the period from 2017 to 2024, have been classified into distinct land-use categories using the proposed IRU-Net model. These categories include built-up areas, bare terrain, snow/ice, clouds, rangelands, trees, flooded vegetation, crops, and areas with no data. The Observations are as follows. Urban areas expanded significantly between 2019 and 2024. Agricultural lands declined, while road networks increased. Vegetation and water bodies remained relatively stable, although minor seasonal variations were observed.

The land-use classifications reflect the changes in landscape that have taken place in this area during the aforementioned period and include important information such as: how much built-up areas increased, tree-covered areas, and water bodies. Its performance to detect and classify these changes reinforces its potential value as an analysis tool for the interpretation of remotely-sensed data over extended periods to facilitate evaluation of land-use trajectories, environmental transitions, or other long-term ecological changes.

The performance divergence between every dataset arises due to the inherent nature of its distinctive features. The dataset is more complex because of the small, irregular shapes of different appearances, which make it difficult to accurately segment. Furthermore, the dataset is unaligned, which makes it harder for the model to identify nuances. However, in both cases, better alignment and richer uniform images boost segmentation score. Part of the differences in performance may also be explained by the different picture formats used: TIFF and colored PNG.

### Visual interpretation

#### Temporal land use changes (2017–2024)

Figure 4 presents classified maps over the Katpadi region from 2017 to 2024:

Observations

- Urban areas expanded significantly between 2019 and 2024.
- Agricultural lands declined, while road networks increased.
- Vegetation and water bodies remained relatively stable, although minor seasonal variations were observed.

Multi-year Methods for Sequential Evolution Processing:

Since here the main focus of this work is on sequential evolution processing, it is essential to provide a clear description of how traditional multi-year methods were used to interpret land-use changes for each transition from 2017 to 2024. Multi-year analysis helps in identifying temporal trends, so correlations and transitions of remote sensing data.

1. *Time series—based analysis* Changes over time in satellite images from 2017 to 2024 were examined using a multi-year approach for gradual or sudden changes in patterns of land use. Analysis of images acquired over certain intervals to detect patterns like the growth of built—up areas, reduction of green cover, and development of water bodies.
  The model was trained to segment and classify land-use patterns at four key time points: 2017, 2019, 2021, and 2024.
  To ensure the model’s robustness, cross-time comparison was employed, where the same geographic regions were analysed across the entire time span, allowing for accurate detection of land cover transitions.
2. *Sequential evolution analysis* The rise and fall of land-use categories were calculated based on the segmented images. As an example, the expansion of built-up areas through the years was mapped, indicating about a 10% increase in land cover classes between 2017 and 2024 along with a larger decline of 7% in rangelands. Analysis for consecutive years was performed using the rate of change (RoC) and temporal change detection algorithms that detect pixel-wise changes to find evolving patterns.

These algorithms enabled a temporal characterisation of the processes involved in urbanisation, deforestation, and water body formations.

### Evaluation metrics

Determined the relevant evaluation criteria, which were precision, recall, Dice coefficient (DSC), IoU, and accuracy from a comprehensive review of related literature. These metrics were used to evaluate the proposed model’s performance on both datasets. The metrics that follow are calculated using the abbreviations TP (true positive), FP (false positive), TN (true negative), and FN (false negative).

1. Dice coefficient (DSC):
  26 $${\text{DSC}} = \frac{{2 \times {\text{TP}}}}{{2 \times {\text{TP}} + {\text{FP}} + {\text{FN}}}}$$
2. Jaccard Coefficient or loU—Intersection Over Union:
  27 $${\text{IoU}} = \frac{{{\text{TP}}}}{{{\text{TP}} + {\text{FP}} + {\text{FN}}}}$$
3. Precision:
  28 $${\text{ Precision }} = \frac{{{\text{TP}}}}{{{\text{TP}} + {\text{FP}}}}$$
4. Recall:
  29 $${\text{ Recall }} = \frac{{{\text{TP}}}}{{{\text{TP}} + {\text{FN}}}}$$
5. Accuracy:
  30 $${\text{Accuracy }} = \frac{{{\text{TP}} + {\text{TN}}}}{{{\text{TP}} + {\text{FP}} + {\text{TN}} + {\text{FN}}}}$$

### Evaluation of cross-dataset performance

Assessing a trained model’s robustness and generalizability requires cross-dataset examination. The variety of datasets makes it possible to assess how well a model works in practical situations and aids in locating and removing biases that may have been in the training set. Identifying the best-performing model requires comparing and evaluating different models via cross-dataset evaluation.

**Fig. 6:**
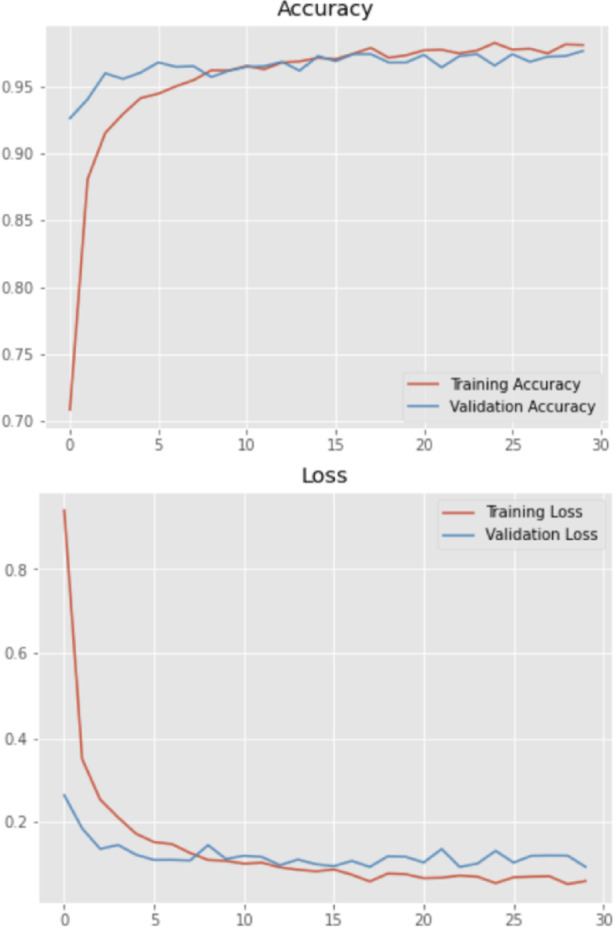
Accuracy and Loss plots for training and validation.

**Fig. 7:**
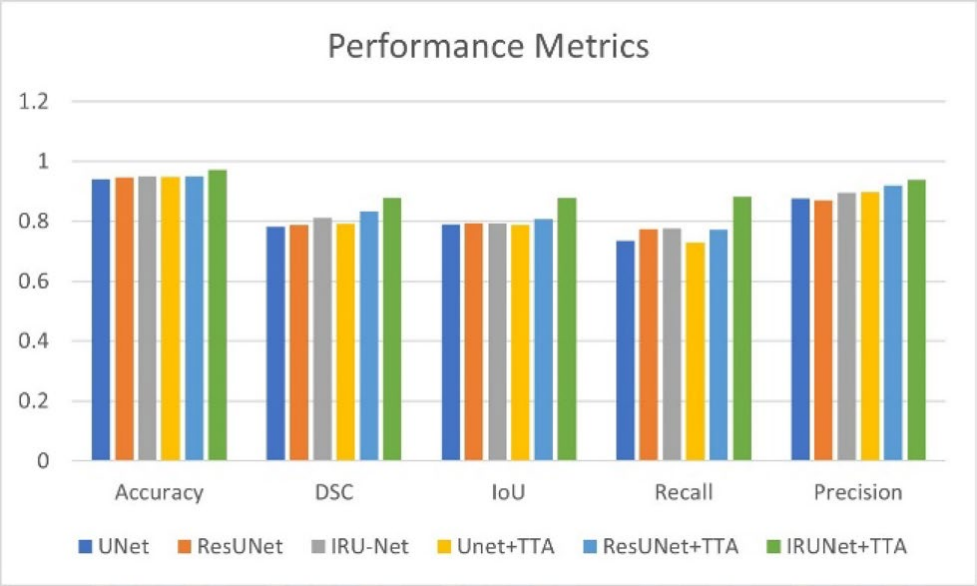
Comparisons of the accuracy, DSC, IoU, recall, and precision of the various models.

Figure 6 presents the accuracy and loss graphs for both the training and validation phases. The outcomes showed that the model had a loss of less than 2% and an accuracy of more than 98%. These metrics demonstrate the model’s superior performance, particularly compared to other models. Figure 7 shows the comparisons of the accuracy, DSC, IoU, recall, and precision of various models, such as UNet, ResUnet, and IRU-Net, with and without the implementation of augmentation. IRUNet captured finer boundaries and small objects (e.g., roads, water bodies) better than UNet and ResUNet. Attention mechanisms in Attention-UNet helped but still missed minor class regions compared to IRUNet.

### Ablation study

To evaluate the effectiveness of the proposed IRU-Net model and the impacts of test time augmentation (TTA), an ablation study comparing UNet, ResUNet, IRU-Net, and their TTA-enhanced equivalents was conducted. Among the performance metrics considered are the Dice similarity coefficient (DSC), accuracy, recall, precision, and intersection over union (IoU). The results are summarized in Table 3.

### Analysis

1. *Baseline models*
  The basic UNet model achieved 93.92% accuracy, 0.7821 DSC, 0.7892 IoU, 0.7349 recall, and 0.8766 precision.
  The ResUNet model performed somewhat better than UNet, with an accuracy of 94.62%, DSC of 0.7875, IoU of 0.7936, recall of 0.7739, and precision of 0.8704.
  The IRU-Net model demonstrated even greater improvement, with an accuracy of 94.90%, a DSC of 0.8109, an IoU of 0.7948, a recall of 0.7768, and a precision of 0.8966.
2. *Impact of TTA*
  The precision increased to 0.8975, the DSC to 0.7912, and the accuracy to 94.81% after TTA and UNet (UNet + TTA) were combined. However, the recall dropped to 0.7296.
  ResUNet + TTA demonstrated a more noticeable improvement, with an accuracy of 95.01%, DSC of 0.8324, IoU of 0.8085, recall of 0.7719, and precision of 0.9192.
  The proposed IRUNet + TTA model demonstrated the greatest improvement over all the other models, with a precision of 0.9471, a recall of 0.8919, an IoU of 0.8896, a DSC of 0.8896, and an accuracy of 98.21%.

To clearly differentiate IRUNet from existing architectures, we provide a comparison in Table 4. IRUNet uniquely combines: InceptionResNetV2 for hierarchical and multi-scale feature extraction. UNet decoder for pixel-level segmentation with skip connections. Multi-level feature fusion at the bottleneck for robust representation. Test-Time Augmentation (TTA) using flipping strategies during inference to improve prediction stability. These architectural innovations enable IRUNet to outperform conventional variants in capturing fine-grained land use features. These architectural innovations enable IRUNet to outperform conventional variants in capturing fine-grained land use features.

**Table 4:** Architecture comparison.

Model	Encoder	Decoder	Feature fusion	TTA	Novel element
UNet	Basic CNN	Yes	No	No	Standard UNet
ResUNet	CNN + Residual	Yes	No	No	Residual connections
Attention-UNet	CNN + Attention Gate	Yes	No	No	Attention in skip paths
IRUNet (ours)	Inception ResNetV2	Yes	Yes	Yes	Multi-scale fusion + TTA

### Model comparisons

Figure 7 visually compares sample segmentations from IRUNet and baseline models:

Analysis:

- IRUNet captured finer boundaries and small objects (e.g., roads, water bodies) better than UNet and ResUNet.
- Attention mechanisms in Attention-UNet helped but still missed minor class regions compared to IRUNet.

The multi-annual class results produced by the proposed IRUNet model not only demonstrate a qualitative gain over baseline methods but also reveal significant spatial and temporal land use relationships in the Katpadi area. One of the evident trends found was the large-scale increase in urban areas, especially from 2019 to 2024, corresponding to established development pressures surrounding Vellore and the expanding role of institutions like VIT. This urban spread has taken place mainly at the cost of agricultural land, a widely documented phenomenon in Indian peri-urban areas. IRUNet correctly outlined these changes, such as the spread of built-up areas into land that was previously farmed.

Moreover, consistent classification of vegetation and water bodies over years demonstrates the ability of the model to cope with seasonal variation, even though temporal sequences were not modeled explicitly. The identification of road networks and thin water streams—usually underperformed by conventional models—was significantly enhanced as a result of the model’s deep feature hierarchy and multi-scale fusion ability. These findings indicate that IRUNet, apart from being highly efficient in classification accuracy, is also sensitive to structural land use change that holds socio-economic and ecological relevance.

The proposed IRUNet consistently outperformed baseline models across all evaluation metrics. The improved land use classification provided by IRUNet has several potential real-world applications: Urban Planning: Monitoring expansion zones and optimizing infrastructure development. Environmental Management: Tracking forest coverage and waterbody preservation efforts. Agriculture: Monitoring seasonal crop land conversion. Disaster Risk Reduction: Identifying vulnerable regions prone to flooding or urban heat islands.

## Conclusion

This study investigates an innovative approach to land use classification by utilizing multi-year Sentinel-2 data and a case study site in the rapidly urbanizing Katpadi region, Vellore District, Tamil Nadu, India. Therefore, our analysis portrays the variations in land usage over a period of time surrounding the Vellore Institute of Technology in these satellite images between 2017 and 2024. In contrast to single models and traditional approaches, our method provides a deep learning-based ensemble network for classifying land cover types that consolidates classification performance and increases robustness through time.

This study proposed IRUNet, a deep learning ensemble combining InceptionResNetV2 and UNet, optimized with Test-Time Augmentation, for land use classification using Sentinel-2 imagery across multiple years. Our model demonstrated superior performance compared to standard architectures, validated through quantitative metrics and visual interpretation.

In this work, we propose an IRU-Net architecture for segmentation. The TTA interface has greatly improved the segmentation performance. The IRU-Net model (InceptionResNet + UNet) Initially, the InceptionResNet is a pretrained model. There is a bridge between those four decoder blocks and four stacked encoder blocks. We segmented the data by averaging the results over multiple phases using TTA. We evaluated the model against suitable models, such as UNet, Attention-UNet, and ResUNets, using satellite datasets.

The IRU-Net + TTA model provided a favorable result compared to other state-of-the-art models, both qualitatively and quantitatively. The IRU-Net + TTA method proves to be highly beneficial in this regard, particularly in situations where there is limited or no research on real-time data collection for clinical investigations.

Although temporal relationships were not explicitly modeled (e.g., using RNNs), sequential year-wise classification still revealed meaningful land use transitions. Future work will focus on incorporating explicit spatiotemporal modeling through Temporal Convolutional Networks (TCN) and ConvLSTM architectures to further enhance predictive capability.

By extracting a wide spectrum of spectral and spatial information from Sentinel-2 images, our method captures dynamic variability in land cover and produces a high-resolution dynamic map of the Earth’s surface. The deep learning-based ensemble network handles combining such different types of data well, which helps the model figure out the complicated connections and interactions between the different types of land cover.

Using large-scale real-world Sentinel-2 multi-year data, we show that our proposed ensemble network achieves state-of-the-art accuracy for land use classification tasks. These results demonstrate the effectiveness of ensemble networks, outperforming single models and traditional classification methods for remote sensing applications.

### Limitations

Our validation is limited to the Katpadi region due to the lack of labeled external datasets. The model treats multi-year data as independent inputs and does not capture temporal dependencies.

### Future work

Extend evaluation to other regions across India and globally. Integrate ConvLSTM or Temporal Convolutional Networks to explicitly model sequential changes in land use. Explore self-supervised pretraining to reduce annotation dependency.

### Real-world applications

The improved land use classification provided by IRUNet has several potential real-world applications:

- *Urban planning* Monitoring expansion zones and optimizing infrastructure development.
- *Environmental management* Tracking forest coverage and waterbody preservation efforts.
- *Agriculture* Monitoring seasonal crop land conversion.
- *Disaster risk reduction* Identifying vulnerable regions prone to flooding or urban heat islands.

## Data Availability

The research data supporting this study have been deposited in IEEE Dataport and are accessible via the following 10.21227/ev8p-r080.
